# Genomic Comparisons Reveal Microevolutionary Differences in *Mycobacterium abscessus* Subspecies

**DOI:** 10.3389/fmicb.2017.02042

**Published:** 2017-10-23

**Authors:** Joon L. Tan, Kee P. Ng, Chia S. Ong, Yun F. Ngeow

**Affiliations:** ^1^Faculty of Information Science and Technology, Multimedia University, Melaka, Malaysia; ^2^Department of Medical Microbiology, Faculty of Medicine, University of Malaya, Kuala Lumpur, Malaysia; ^3^Department of Pre-clinical Sciences, Faculty of Medicine and Health Sciences, Universiti Tunku Abdul Rahman, Petaling Jaya, Malaysia

**Keywords:** non-tuberculous mycobacterium, *Mycobacterium abscessus* subspecies, microevolution, recombination, positive selection

## Abstract

*Mycobacterium abscessus*, a rapid-growing non-tuberculous mycobacterium, has been the cause of sporadic and outbreak infections world-wide. The subspecies in *M. abscessus* complex (*M. abscessus, M. massiliense*, and *M. bolletii*) are associated with different biologic and pathogenic characteristics and are known to be among the most frequently isolated opportunistic pathogens from clinical material. To date, the evolutionary forces that could have contributed to these biological and clinical differences are still unclear. We compared genome data from 243 *M. abscessus* strains downloaded from the NCBI ftp Refseq database to understand how the microevolutionary processes of homologous recombination and positive selection influenced the diversification of the *M. abscessus* complex at the subspecies level. The three subspecies are clearly separated in the Minimum Spanning Tree. Their MUMi-based genomic distances support the separation of *M. massiliense* and *M. bolletii* into two subspecies. Maximum Likelihood analysis through dN/dS (the ratio of number of non-synonymous substitutions per non-synonymous site, to the number of synonymous substitutions per synonymous site) identified distinct genes in each subspecies that could have been affected by positive selection during evolution. The results of genome-wide alignment based on concatenated locally-collinear blocks suggest that (a) recombination has affected the *M. abscessus* complex more than mutation and positive selection; (b) recombination occurred more frequently in *M. massiliense* than in the other two subspecies; and (c) the recombined segments in the three subspecies have come from different intra-species and inter-species origins. The results lead to the identification of possible gene sets that could have been responsible for the subspecies-specific features and suggest independent evolution among the three subspecies, with recombination playing a more significant role than positive selection in the diversification among members in this complex.

## Introduction

*Mycobacterium abscessus* is a rapidly growing species of non-tuberculous mycobacterium (NTM). In 2011, this species was divided into two subspecies, *M. abscessus* subsp. *abscessus* and *M. abscessus* subsp. *bolletii* (Leao et al., 2011) but subsequent comparative studies including those based on whole genome sequencing (Cho et al., 2013; Tan et al., 2015) supported division of the *M. abscessus* complex into three subspecies, *M. abscessus* subsp. *abscessus, M. abscessus* subsp. *bolletii*, and *M. abscessus* subsp. *massiliense*. In this paper, the subspecies are referred to as *M. abscessus, M. bolletii*, and *M. massiliense*. All three subspecies are opportunistic pathogens associated with significant morbidity, particularly in patients with cystic fibrosis (Olivier et al., 2003; Jönsson et al., 2007; Levy et al., 2008; Bryant et al., 2013; Davidson et al., 2013). Although genetically closely related, they show differences in drug resistance (Koh et al., 2011, 2013; Mitra et al., 2012; Griffith, 2014), association with clinical disorders (Koh et al., 2011; Stout and Floto, 2012; Roux et al., 2015), and mode of transmission (Bryant et al., 2013; Tettelin et al., 2014). To date, the evolutionary forces that could have contributed to these biological and clinical differences are still unclear.

Recombination, an important evolutionary force for the diversification of bacterial core genomes (Ehrlich et al., 2005; Redfield et al., 2006), has been reported in the *M. abscessus* complex (Sassi et al., 2014). Gene acquisition at the hypothetical common ancestor apparently played an important role in the evolution of this species complex and the enhanced virulence in some members could have come from their ability to acquire genes from different bacterial genera via horizontal transfer.

Positive selection is another evolutionary force that contributes to significant selective advantage in organisms, varying from prokaryotes to eukaryotes (Petersen et al., 2007; Lefébure and Stanhope, 2009; Cagliani and Sironi, 2013). For instance, the insensitivity of *M. tuberculosis* to most common antibiotics has been hypothesized to be due to convergent evolution as a result of positive selection on drug resistance-associated genes (Casali et al., 2012; Farhat et al., 2013). In some cases, positive selection acts together with horizontal transfer and homologous recombination to bring about bacterial diversification (Donati et al., 2010).

In this study, we used bioinformatic analysis on 243 genomes to understand how the microevolutionary processes of homologous recombination and positive selection influenced the diversification of the *M. abscessus* complex at the subspecies level.

## Materials and Methods

### Genome Sequences

Of the 243 genomes examined, 12 were from clinical strains isolated between 2009 and 2011 in the Clinical Microbiology Laboratory of University of Malaya Medical Centre (UMMC), Kuala Lumpur, Malaysia. These strains were subjected to whole genome shotgun sequencing using the Illumina Genome Analyzer IIX platform, as described previously (Tan et al., 2013). For the remaining genomes, we downloaded their whole genome sequences from the NCBI ftp Refseq database (March 2016) (**Supplementary Table S1**). We included strains from different parts of the world to increase the likelihood of identifying subspecies-specific characteristics instead of just clonal traits.

### Genome-Wide Intra-subspecies and Inter-subspecies Comparisons

For *in silico* inter-subspecies and intra-subspecies comparisons, a single pseudo-contig for each of the 243 genomes was generated by using “NNNNNCACACACTTAATTAATTAAGTGTGTGNNNNN” as a linker between contigs. The insertion of this 36 bp pseudo-molecule introduces a stop codon in any of the six open reading frames, and hence will not affect the annotation of each genome (Tettelin et al., 2005; Xu et al., 2010; Peng et al., 2017). The maximal unique matches index (MUMi) was implemented to infer the genomic relatedness among the strains. The choice of MUMi over the more established average nucleotide identity (ANI) is because of its greater robustness for the inference of intra-species divergence and its inclusion of both core and accessory genome sequences for the calculation of divergence. Hence, NUCmer of MUMmer was used to perform the pairwise genome alignments (Kurtz et al., 2004). Matches present with the effect of DNA inversion events were identified and overlapping segments of unique matches were filtered. The resulting data was used as input in the MUMi perl script (Deloger et al., 2009). A MUMi value of 0 indicates identical genome sequences whereas the maximum value of 1 indicates the most distantly related genomes.

### Defining Orthologous Sequences and Alignments

The genome sequences were annotated using locally installed GeneMarkS with default parameters (Besemer et al., 2001). The predicted protein sequences were clustered using ProteinOrtho (Lechner et al., 2011). Protein sequences with minimum e-value of 1 × 10^-5^, alignment coverage of 50% and clustering connectivity of 0.1 were categorized into a single protein family (Lechner et al., 2011). Sequences were then extracted based on the defined families. Multiple-aligned sequences were obtained in fast Fourier transform (FFT) algorithm as implemented in MAFFT, using FFT-NS-I as the iterative refinement method (Katoh et al., 2002). The alignment of corresponding codons was achieved in PAL2NAL (Suyama et al., 2006).

### Identification of Genes under Positive Selection

We examined each of the non-duplicated conserved proteins for positive selection in each of the three subspecies, using Branch-site model (model = 2; NS sites = 2) in CodeML of PAML on the dataset (Yang, 2007). In the Branch-site model, foreground branches comprised the branches of interest to be compared with the rest of the branches on a phylogenetic tree (background branches) (Zhang et al., 2005). Two models (null and alternate) were compared to select a suitable mode to depict the evolution of the genes. In the null model, codons may evolve neutrally or under purifying selection (fix_omega = 1, omega = 1) in both background and foreground branches. In the alternate model, codons were assumed to be undergoing positive selection in foreground branches but not in background branches (fix_omega = 0, omega = 1). The *p*-value was derived from the likelihood ratio test (LRT), assuming Chi-square distribution with degree of freedom of 1 (Yang, 1998). Genes were treated as undergoing positive selection if *p*-value is less than 0.01, and the non-synonymous to synonymous mutation ratio (dN/dS) is greater than one. The genes with significant traces of positive selection were further analyzed with the Bayes Empirical Bayes (BEB) method, to retrieve the positions of amino acids on the genes that were substituted owing to selection forces (Yang et al., 2005). The genes supported in BEB were subjected for subcellular localization prediction in CELLO webserver (Yu et al., 2006).

To investigate whether the dN/dS of each of the identified positively selected sequences was influenced by recombination, we performed recombination detection based on the three robust general testing methods (Brown et al., 2001; Posada et al., 2002), namely Neighbor Similarity Score (NSS) (Jakobsen and Easteal, 1996), Maximum χ^2^ (Max χ^2^) (Smith, 1992), and Pairwise Homoplasy Index (PHI) (Bruen et al., 2006). NSS is based on clustering of compatibilities of the informative adjacent sites in the form of a matrix; PHI highlights the calculation of minimum number of homoplasies without the influence of the sample’s history, and Max χ^2^ compares putative recombination break-points in two sequences, with other sequences within an alignment.

### Recombination Analysis

The genomes were aligned based on locally-collinear blocks (LCBs) as defined by the progressiveMauve algorithm (Darling et al., 2010). Owing to the computational limitation in the handling of a large dataset comprising 243 genomes with a mean size of 5.1 M base pairs, we applied the following protocol – (1) 15 genomes each from *M. abscessus* and *M. massiliense* were selected as representatives for comparisons. The selection was based on the mean MUMi (indicating the average genomic distance between paired strains in a group), with five genomes each from the strains with the highest, median and lowest mean MUMi values. The intention of the selection was to maximize the inclusion of strains with high genetic diversities. Since there were only 16 *M. bolletii* strains, all *M. bolletii* genomes were included for the genome-wide alignments (**Supplementary Table S1**). (2) Only the LCBs with a minimum size of 500 bp which were shared by all the strains were used for the analysis. An in-house Biopython script was written to separate each LCB from the XMFA alignment file. (3) The sequence of each LCB was then identified from the genomes that were not used in the Mauve alignment. Each of the resulted LCB was manually curated to remove artifacts, and concatenated to form super-sequences. The super-sequences and a PhyML generated super-sequences SNPs tree were subjected to recombination study in the ClonalFrameML (Didelot and Wilson, 2015).

## Results

### Population Structure

**FIGURE 1:**
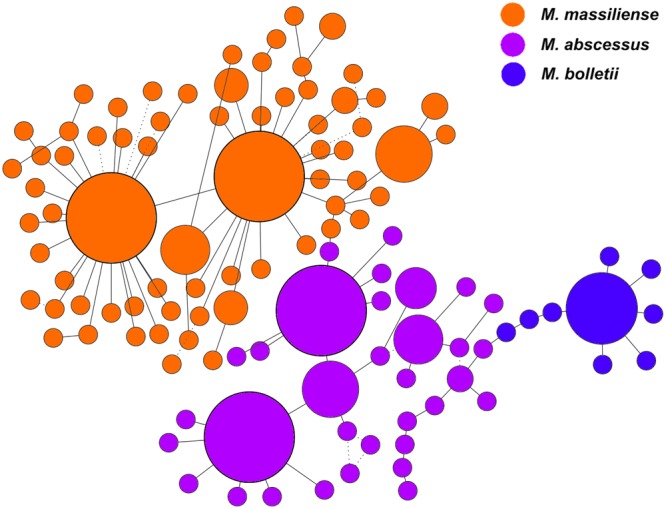
Minimum spanning tree based on 50 informative genes of the *M. abscessus* complex. The size of each circle corresponds to the number of genomes within respective nodes.

The genomes used in this study comprised the three known subspecies of *M. abscessus* complex isolated from multiple countries. The subspecies for each of the 243 strains was identified *in silico* using the concatenation of the 50 proposed informative genes previously reported (Tan et al., 2013). The resulting pseudo-sequence was used in the calculation of a minimum-spanning tree (MST) (**Figure 1**). The MST illustrates the relationships of data based on the shortest possible distance between two data points. Strains from the same subspecies would have low dissimilarities and short genetic distances from each other, and hence, are expected to form a cluster. Based on this classification, 16 strains in our study clustered as *M. bolletii*, 138 as *M. massiliense*, and 89 as *M. abscessus*.

On the MST, the size of each circle corresponds to the number of genomes within respective nodes. Generally, strains within the same subspecies were clustered together without passing through any node from other subspecies. The MST was constructed with 100 bootstrap replications and each bootstrapping tree was consistently observed to have 110 nodes. All edges were supported with high bootstrap replication values at a minimum of 90% (represented in solid lines), except for 11 edges with lower bootstrap values of 50–70% (represented in dotted lines). The lower confidence edges were distant from the edges connecting the different subspecies. Hence, there were no strains with questionable subspecies status. No sign of geographical clustering among the strains was observed. The MST also highlighted the greater diversity among *M. massiliense* strains compared to *M. abscessus* and *M. bolletii*. These observations suggest that the evolution of the *M. abscessus* complex is not clonal but is driven by the genetic characteristics of each subspecies within the complex.

### Genome-Wide Inter- and Intra-subspecies Comparisons

Using MUMi to define genomic distances, the three subspecies showed, as expected, shorter intra-subspecies distances compared to inter-subspecies distances (**Supplementary Table S2**). The inter-subspecies interquartile range for *M. bolletii* – *M. massiliense, M. bolletii* – *M. abscessus*, and *M. abscessus* – *M. massiliense* were 0.2326, 0.2072, and 0.2222, respectively. Hence, the genomic distances between *M. massiliense* and *M. bolletii* were longer than the distances between *M. massiliense* and *M. abscessus* or *M. bolletii* and *M. abscessus*. The taxonomic separation of *M. massiliense* from *M. bolletii* has been proposed by several research groups based on the analysis of core sequences with ANI as well as linear and network phylogenies (Cho et al., 2013; Tan et al., 2013, 2015). Our analysis of genomic distances lends further support, from a different perspective, for the separation of the two subspecies.

**FIGURE 2:**
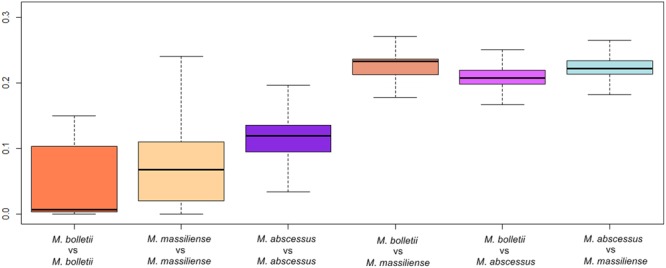
Maximum Unique Matches index (MUMi) of *M. abscessus* subspecies represented in boxplots. The heavy line represents the median MUMi value.

The intra-subspecies interquartile range of MUMi was 0.0998 in *M. bolletii*, 0.0898 in *M. massiliense* and 0.0407 in *M. abscessus*. From the boxplot constructed (**Figure 2**), the MUMi of *M. bolletii* skewed toward the higher values of the box, reflecting a relatively conserved genetic content in this subspecies. In contrast, the MUMi of *M. abscessus* was skewed toward the lower values in the boxplot, indicating that the majority of the *M. abscessus* strains have longer genomic distances from each other. The MUMi of *M. massiliense*, however, showed a symmetrical distribution, indicating the presence of roughly equal numbers of conserved and diverse strains.

### Positive Selection

Our Maximum Likelihood analysis through dN/dS calculation on each of the 1,090 non-paralogous core genes in our dataset supports the presence of genes that have undergone selective pressure. It has been said that the dN/dS ratio is not sensitive enough to detect gene sets that have undergone positive selection among closely related species (Kryazhimskiy and Plotkin, 2008). However, based on the ratios of mutations that were supported in the Likelihood Ratio test (Yang, 1998), we managed to uncover eight genes in *M. massiliense*, 14 in *M. abscessus* and 18 in *M. bolletii*, that could have undergone positive selection (*p*-value < 0.01), during the course of divergence among the three subspecies.

Based on the results of the three independent recombination methods (NSS, PHI, and Max χ^2^) and a *p*-value cutoff at 0.01, only four out of 18 positively selected genes in *M. bolletii* (**Supplementary Table S3**) and 2 out of 14 genes in *M. abscessus* (**Supplementary Table S4**) were identified as having undergone recombinations. None of the positively selected genes in *M. massiliense* (**Supplementary Table S5**) showed evidence of recombination.

Bonferroni correction is commonly applied to investigate the possibility of including genes with type I errors (false-positive) in positive selection analyses (Wong et al., 2004; Yang, 2007). On the other hand, it is also known to cause false negatives when used on a large number of tests (Perneger, 1998). In the current study, after performing Bonferroni correction for multiple testing, only two genes in *M. massiliense*, three genes in *M. abscessus* and eight genes in *M. bolletii* were left with a significant *p*-value for positive selection. However, the genes with an insignificant *p*-value after adjustment also failed in the recombination tests, indicating that they are likely to be false negatives in the Bonferroni test, and that the substitutions observed within them can still be the result of selection rather than random mutation or recombination.

#### Positive Selection in *M. massiliense*

The positively selected genes identified in this subspecies are an isochorismatase, a beta-lactamase-like protein, a probable deoxyribonuclease tatD, a thymidylate kinase Tmk, 30S ribosomal protein S3 and three hypothetical proteins. Metal ion acquisition is essential for bacterial invasion in the mammalian host and isochorismatase is one of the important enzymes for siderophore-based ferric iron acquisition by cells (Miethke and Marahiel, 2007). The association of this protein with virulence has been reported in *Acinetobacter baumannii* (Gebhardt et al., 2015). The twin-arginine translocation (tat) system has been shown to play an important role in the virulence of *Yersinia pseudotuberculosis* (Lavander et al., 2006) and has been associated with *E. coli* meningitis (Siddiqui et al., 2012). Although the function of the tatD component is not fully known, it has been reported to be the virulence factor in *Plasmodium* and a potential vaccine candidate for malaria (Chang et al., 2016). We had previously reported thymidylate kinase and 30S ribosomal protein S3 as being suitable for the distinction of *M. massiliense* from other mycobacteria including other subspecies of the *M. abscessus* complex (Tan et al., 2015). The positive selection of these genes implies that they may have advantageous biological functions besides being suitable markers for classification.

#### Positive Selection in *M. bolletii*

Of the 11 positively selected proteins with known functions, two affect bacterial growth, i.e., phosphoribosylaminoimidazole-succinocarboxamide synthase purC which has been described to affect the growth of *M. tuberculosis* (Brown et al., 2005) and Fructose-bisphosphate aldolase, frequently reported to be important for growth in pathogens such as *M. tuberculosis* and multidrug-resistant *Staphylococcus aureus* (Capodagli et al., 2014; Puckett et al., 2014). The remaining nine proteins are associated with cell membrane entry (mce), transcription regulation (IclR family), reaction to heat shock (Hsp20), metabolic reactions (*trans-*aconitate methyl transferase, 5-methyltetrahydropteroyltriglutamate–homocysteine methyltransferase), and probable endonuclease activity (as described for protein S16 in the 30S ribosomal subunit, by Oberto et al., 1996).

Despite its reputation as a multiple antibiotic resistant NTM, we could only find one positively selected gene associated with antibiotic resistance in this subspecies, i.e., a transmembrane efflux protein previously reported in mycobacteria (De Rossi et al., 2006). This observation supports the concept that antibiotic resistance incurs a fitness cost in bacteria in the absence of antibiotic selective pressure, and hence, is not likely to be positively selected during bacterial speciation or subspeciation.

#### Positive Selection in *M. abscessus*

In *M. abscessus*, positive selection was observed in the pyridoxine5-phosphate oxidase gene shown to be involved in Vitamin B6 biosynthesis (Dick et al., 2010; Mashalidis et al., 2011), the acyl-CoA thioesterase II gene associated with drug resistance expression in mycobacteria (Danelishvili et al., 2005), transcriptional regulator proteins including the IclRfamily, putative transcriptional regulator, and tetR family transcriptional regulator (Ramos et al., 2005). Also positively selected are four hypothetical proteins, one of which was also identified together with a probable regulator protein in *M. bolletii*, and the tatD and 30S ribosomal protein S3 that were also found in *M. massiliense*.

### Site-Specific Positive Selection

In each *M. abscessus* subspecies there is evidence of selective pressure on specific positions in proteins. Manual inspection of sequence alignments and substitutions showed the involvement of 13 amino acids from two proteins in *M. abscessus*, 24 amino acids from five proteins in *M. massiliense* and 11 amino acids from four proteins in *M. bolletii*. The amino acids exerting site-specific positive selection were statistically evaluated with BEB probability, with 50–100% confidence (**Table 1**).

**Table 1:** List of positively selected amino acids specific to each of the subspecies.

Protein	Position	Original amino acid	Substituted amino acid	BEB probability (%)	Subspecies
Name: 30S ribosomal protein S3 Function: binds the lower part of the 30S subunit head. Binds mRNA in the 70S ribosome, positioning it for translation. Subcellular localization: cytoplasmic	217 220 222 223	S A P A	A E G R	75 96 98 99	*M. abscessus* M. *abscessus* M. *abscessus* M. *abscessus*
Name: pyridoxine 5-phosphate oxidase Function: catalyzes the oxidation of either pyridoxine 5′-phosphate (PNP) or pyridoxamine 5′-phosphate (PMP) into pyridoxal 5′-phosphate (PLP) Subcellular localization: cytoplasmic	86 142 172 199 200 201 202 203 204	A A E E N L S L P	T G D G P V T A L	82 88 84 85 99 87 88 99 85	*M. abscessus* M. *abscessus* M. *abscessus* M. *abscessus* M. *abscessus* M. *abscessus* M. *abscessus* M. *abscessus* M. *abscessus*
Name: MAB_2825 hypothetical protein Function: uncharacterized Subcellular localization: membrane	60 109 410 412	K I G P	R V K A	70 71 92 72	*M. massiliense* M. *massiliense* M. *massiliense* M. *massiliense*
Name: 30S ribosomal protein S3 Function: binds the lower part of the 30S subunit head. Binds mRNA in the 70S ribosome, positioning it for translation. Subcellular localization: cytoplasmic	259 260 261 262 263 265 268 269 270 271	P A S D G S S A E T	G G N T S E A E T S	99 84 100 99 99 100 79 81 99 77	*M. massiliense* M. *massiliense* M. *massiliense* M. *massiliense* M. *massiliense* M. *massiliense* M. *massiliense* M. *massiliense* M. *massiliense* M. *massiliense*
Name: thymidylate kinase Tmk Function: phosphorylation of dTMP to form dTDP in both *de novo* and salvage pathways of dTTP synthesis Subcellular localization: cytoplasmic	20 21 23 24 26 27	E K I A G N	R A T R S I	100 98 74 98 99 63	*M. massiliense* M. *massiliense* M. *massiliense* M. *massiliense* M. *massiliense* M. *massiliense*
Name: isochorismatase hydrolase Function: involved in isochorismatase metabolism Subcellular localization: cytoplasmic	209 211	T A	S E	84 98	*M. massiliense* M. *massiliense*
Name: beta-lactamase -like protein Function: unknown. Hypothetically involved in drugs resistant Subcellular localization: cytoplasmic	242 243	N T	S K	52 84	*M. massiliense* M. *massiliense*
Name: MAB_1249 hypothetical protein Function: uncharacterized Subcellular localization: cytoplasmic	31 172	G Y	S S	66 64	*M. bolletii M. bolletii*
Name: cytochrome P450 Function: oxidoreductase activity Subcellular localization: cytoplasmic	56 93 275 351	K M E A	R L A G	57 60 73 67	*M. bolletii M. bolletii M. bolletii M. bolletii*
Name: MAB_2993C hypothetical protein Function: amino acid transmembrane transporter activity Subcellular localization: membrane	301	R	T	68	*M. bolletii*
Name: fructose-bisphosphate aldolase Function: glycolytic process Subcellular localization: cytoplasmic	150 154 157 160	A S V K	Q A L H	99 97 56 96	*M. bolletii M. bolletii M. bolletii M. bolletii*

*BEB, Bayes Empirical Bayes.*

### Genome-Wide Recombinations

**FIGURE 3:**
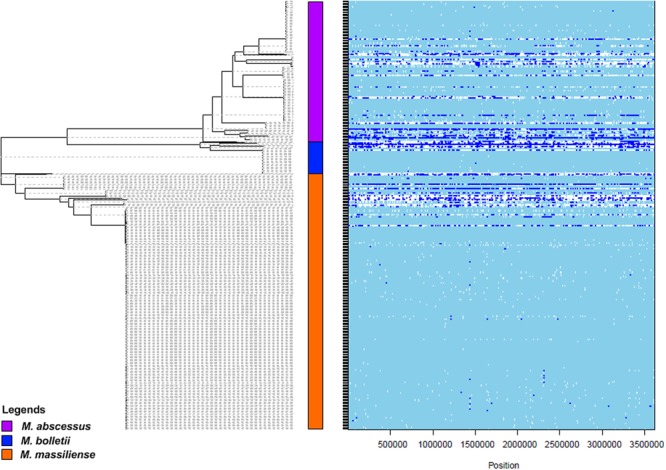
Genome wide recombination plot (obtained with the ClonalFrameML) and supersequence SNP-based phylogenetic tree (generated with PhyML) for the *M. abscessus* complex. The plot on the right shows the presence of recombination (in blue) and mutation (in white) throughout the genomes of the *M. abscessus* complex. In the phylogenetic tree, purple = *M. abscessus*, red = *M. bolletii*, blue = *M. massiliense*.

Apart from Darwinian evolution, recombination has been proposed as the alternative event for evolution (Livingstone and Rieseberg, 2004; Barton, 2010). Recent phylogenomics network analysis showed reticulations between and within the subspecies of *M. abscessus* complex, suggesting the occurrence of recombination events (Tan et al., 2015). In this study, we investigated the genome-wide recombinations that are present in both coding and non-coding regions of the *M. abscessus* complex, to better understand the genomic properties that could have facilitated the emergence of *M. abscessus* complex as a successful opportunistic pathogen. Overall, 1,468 recombination events (excluding the putative ancestral nodes) were detected from the 1,407 LCBs generated by progressiveMauve. The lengths of recombinations ranged from 2 to 34,082 bp. The ratio of rates at which polymorphisms were introduced by recombination and mutation was R/theta = 0.14, whereas the ratio of recombination and mutation (r/m) was 1.99. Hence, recombination caused two times more polymorphism than mutation. From the illustration in **Figure 3**, extensive recombination was observed in the three putative ancestral nodes leading to the three subspecies. Since there are long evolutionary distances separating the three putative ancestral nodes, it is not known whether the accumulation of the recombinations occurred after the subspeciation event or was responsible for the independent emergence of the three subspecies.

Besides computing the whole population genome-wide recombination structures, subspecies- specific recombinations were also investigated by r/m estimations. The results suggest a higher recombination impact among *M. massiliense* (r/m = 2.17), with almost equal effect of substitutions and mutations in *M. abscessus* (r/m = 1.41) and *M. bolletii* (r/m = 0.95). We predicted 675 recombination events in *M. abscessus*, 387 in *M. bolletii* and 786 in *M. massiliense*. The analysis on recombination segments identified the origins of approximately 23% of total recombination segments in *M. abscessus* (intrasubspecies origin – 103; intersubspecies origin – 54), 16% in *M. bolletii* (intrasubspecies origin – 16; intersubspecies origin – 44) and 17% in *M. massiliense* (intrasubspecies origin – 57; intersubspecies origin – 80). There were 33 segments in *M. abscessus*, 4 in *M. bolletii* and 11 in *M. massiliense* that could not be confirmed to be from an intra- or inter- subspecies origin. The segments that could not be found within the *M. abscessus* complex might have been acquired from foreign origins. However, we did not find insertion sequences (ISs) that could have contributed to the recombination events found in the *M. abscessus* complex. Neither did we find any CRISPRs that could have prevented the entry of foreign genetic material at the recombination sites. It is possible that novel genetic mechanisms are involved in these recombination events.

## Discussion

Speciation genomics has contributed immensely to the understanding of bacterial evolution and diversity (Seehausen et al., 2014). It has enabled the identification of genetic features that are impossible to define with conventional gene-based inference analysis (Zhou et al., 2013; Librado et al., 2014), particularly in bacterial genera with very closely related members such as *Salmonella* variants (Desai et al., 2013) and drug-resistant *Mycobacterium tuberculosis* strains (Farhat et al., 2013). In this study, we focused on members of the *M. abscessus* complex which differ in their drug susceptibility and invasiveness, with *M. massiliense* reported to be the least associated with antibiotic resistance but the most frequently isolated from clinical material, and *M. bolletii* being the most frequently associated with multiple antibiotic resistance but the least frequently isolated from clinical specimens (Koh et al., 2011; Aitken et al., 2012; Nessar et al., 2012; Harris and Kenna, 2014; Tettelin et al., 2014). We tried to look for evidence of evolutionary trends to explain these differences.

The MST showed the strains to be clustered according to the subspecies they belong to rather than by their countries of origin. Within the *M. massiliense* cluster, there is a wider spread of genotypes than in the other two subspecies. This feature indicates higher allelic variability among *M. massiliense* genomes. In contrast, *M. bolletii* showed the least allelic variation. Supported by our data on MUMi evaluation of both core and accessory genomes, the low median intra-subspecies index indicated that *M. bolletii* is conserved compared to *M. massiliense* and *M. abscessus*. This could be reflected in its low prevalence in infections. Using the same data, the inter-species distance appears longer between *M massiliense* and *M. bolletii* than between either of them and *M. abscessus*. This provides another reason to consider *M. massiliense* and *M. bolletii* as distinct subspecies.

The impact of the accessory genome on the evolution of the *M. abscessus* complex has been discussed by other researchers (Choo et al., 2014; Sassi et al., 2014). In this analysis, we focused on the evolutionary pressure within the core genomes of the complex. We found indications of positive selection in a small proportion of genes in all three subspecies. These genes encode enzymes important for the regulation of bacterial growth, metabolism and replication, as well as proteins for virulence, drug resistance and reaction to external stimuli. There is no obvious correlation between subspecies and the function of genes positively selected. The selection of genes for pathogenicity and drug resistance in all three subspecies reflects the evolutionary adaptation of these environmental bacteria to their role as human and animal pathogens.

From the ratio of synonymous and non-synonymous mutations and the evaluation of amino-acid changes based on BEB, we speculated site-specific positive selection resulting in amino acid substitutions. The biological significance of these substitutions is not known, but two proteins involved, the 30S ribosomal protein S3 and thymidylate kinase, were previously found to contain specific signatures for the classification of *M. massiliense* (Tan et al., 2015). We would like to propose that the positively selected proteins that we identified in a systematic way, using sequence data from 243 genomes, would be valuable for the classification of the *M. abscessus* complex.

Our overall results suggest that the evolution of the *M. abscessus* complex is driven more by recombination than positive selections. Interestingly, our data revealed three independent recombination landscapes in the three subspecies. In a previous study (Sapriel et al., 2016), the impact of recombination in *M. abscessus* was the highest among the three subspecies. In the current study, *M. massiliense* appeared to be the most highly affected by recombination, followed by *M. abscessus* and *M. bolletii*, in descending order. The difference could be due to the use of different datasets. Sapriel et al. (2016) studied recombination in the single-targeted gene sets whereas we studied genome-wide recombination landscapes. The higher impact of recombination in *M. massiliense* compared to that in *M. abscessus* is also supported by our previous network phylogenomics analysis (Tan et al., 2015). Nevertheless, the consensus from both Sapriel et al. (2016) and our study is that recombination serves as the driver for the *M. abscessus* complex evolution for pathoadaptation. Lefébure and Stanhope (2007) suggested that recombination contributes to faster adaptation when positive selection is too slow. A high level of genetic similarity has been reported among clinical *M. massiliense* strains isolated from different countries (Davidson et al., 2013; Tettelin et al., 2014) and this similarity has been used to postulate person-to-person transmission among cystic fibrosis patients (Bryant et al., 2013). On the other hand, the genetic similarity among clinical *M. massiliense* strains could also be the result of horizontal transfer and recombination involving common genetic determinants for infectivity. This could illustrate the advantage conferred by a high recombination rate on the pathoadaptation of *M. massiliense*.

## Conclusion

Our findings suggest that the diversity of the *M. abscessus* complex has been shaped by recombination more than Darwinian selection, and that each subspecies of the complex appears to have evolved independently. The proteins we identified to have undergone positive selection and recombination are expected to facilitate future studies on the functional significance of proteins in the *M. abscessus* complex.

## Author Contributions

Planned and conceived the experiments: JLT and YFN. Performed the experiments: JLT and YFN. Analyzed the experiments: JLT, KPN, CSO, and YFN. Drafted the manuscript and contributed to the data interpretation: JLT, KPN, CSO, and YFN. All authors read, critically revised and approved the final manuscript.

## Conflict of Interest Statement

The authors declare that the research was conducted in the absence of any commercial or financial relationships that could be construed as a potential conflict of interest.

## Supplementary Material

TABLE S1 List of strains used in the study.

TABLE S2 Pairwise Maximal Unique Matches Index (MUMi) of the *M. abscessus* complex.

TABLE S3 List of positively selected genes in *M. bolletii*.

TABLE S4 List of positively selected genes in *M. abscessus*.

TABLE S5 List of positively selected genes in *M. massiliense*.
